# 5-aminoimidazole-4-carboxamide ribonucleotide-transformylase and inosine-triphosphate-pyrophosphatase genes variants predict remission rate during methotrexate therapy in patients with juvenile idiopathic arthritis

**DOI:** 10.1186/1546-0096-12-S1-P10

**Published:** 2014-09-17

**Authors:** Serena Pastore, Gabriele Stocco, Valentina Moressa, Chiara Sandrin, Giuliana Decorti, Loredana Lepore, Andrea Taddio

**Affiliations:** 1 University of Trieste, Institute for Maternal and Child Health, IRCCS Burlo Garofolo, Italy; 2 Department of Life Sciences, Italy; 3 University of Trieste, Italy; 4 Institute for Maternal and Child Health , IRCCS Burlo Garofolo, Italy; 5 University of Trieste, Institute for Maternal and Child Health , IRCCS Burlo Garofolo, Trieste, Italy

## Introduction

For children with Juvenile Idiopathic Arthritis (JIA) who fail to respond to methotrexate, the delay in identifying the optimal treatment at an early stage of disease can lead to long-term joint damage. Recent studies indicate that relevant variants to predict methotrexate response in JIA are those in 5-aminoimidazole-4-carboxamide ribonucleotide-transformylase (ATIC), inosine-triphosphate-pyrophosphatase (ITPA) and solute-liquid-carrier-19A1 (SLC19A1) genes.

## Objectives

The purpose of the study was to explore the role of these candidate genetic factors on methotrexate response in an Italian cohort of children with JIA.

## Methods

Clinical response to methotrexate was evaluated clinical remission stable for a 6-months period, as ACRPed score and as change in JADAS score. The most relevant SNPs for each gene considered were assayed on patients’ DNA. ITPA activity was measured in patients’ erythrocytes.

## Results

69 patients with JIA were analyzed: 52.2% responded to therapy (ACRPed70 score), while 37.7% reached clinical remission stable for 6 months. ATIC rs2372536 GG genotype was associated with improved clinical remission (adjusted p-value = 0.0090). For ITPA, rs1127354 A variant was associated with reduced clinical remission: (adjusted p-value = 0.028); this association was present even for patients with wild-type ITPA and low ITPA activity.

## Conclusion

Genotyping of ATIC rs2372536 and ITPA rs1127354 variants or measuring ITPA activity could be useful to predict methotrexate response in children with JIA after validation by further prospective studies on a large patient cohort.

## Disclosure of interest

None declared.

